# Preoperative serum C-reactive protein levels and postoperative survival in patients with esophageal squamous cell carcinoma: a propensity score matching analysis

**DOI:** 10.1186/s13019-019-0981-0

**Published:** 2019-09-18

**Authors:** Wei Huang, Leilei Wu, Xuan Liu, Hao Long, Tiehua Rong, Guowei Ma

**Affiliations:** The Department of Thoracic Surgery, Sun Yat-sen University Cancer Center, Guangdong Esophageal Cancer Institute, State Key Laboratory of Oncology in South China, 651 Dongfengdong Road, Guangzhou, Post Code: 510060 China

**Keywords:** C-reactive protein, Curative resection, Esophageal cancer, Cutoff value, Survival analysis, Propensity scored matching

## Abstract

**Objectives:**

This study tested the relationship between preoperative serum C-reactive protein (CRP) levels and cancer-specific prognosis in patients with esophageal squamous cell carcinoma who have undergone curative resection.

**Methods:**

We conducted a retrospective study on 961 patients with esophageal squamous cell cancer who underwent curative esophagectomy from 2006 to 2012 at the Sun Yat-sen University Cancer Center. Preoperative serum CRP levels were determined, and a cutoff value of 5.0 mg/mL was established. Propensity score matching (PSM) was performed to reduce the selection bias between patients with low CRP (≤ 5.0 mg/mL) and those with high CRP (> 5.0 mg/mL) levels based on age, tumor-lymph node-metastasis (TNM) stage, and tumor grade. The prognostic value of preoperative CRP levels was determined using life table, Kaplan–Meier, and Cox proportional hazards analyzes.

**Results:**

In the unmatched cohort, the 3-year and 5-year survival rates were 57 and 53%, respectively, in patients with high preoperative CRP levels (> 5.0 mg/mL) and 68 and 56%, respectively, in those with low preoperative CRP levels (≤ 5.0 mg/mL). The difference in the survival rates of the 2 groups was significant (*p* = 0.004). Univariate survival analysis revealed that the preoperative CRP levels, TNM stage, tumor grade, drinking history, and anastomosis method were prognostic factors for overall survival (OS). Before conducting PSM, the low-CRP group had a lower age (*p* = 0.001), lower histological grade (*p* = 0.086), and lower TNM stage (*p* = 0.254).

After PSM, 176 patients with low CRP levels and 176 of those with high CRP levels were enrolled in the analysis. In the matched cohort, the 3-year and 5-year survival rates were 56 and 50%, respectively, in patients with high preoperative CRP levels (> 5.0 mg/mL) and 68 and 56%, respectively, in those with low preoperative CRP levels (≤ 5.0 mg/mL). The difference in the survival rates between the low- and high-CRP groups was significant (*p* = 0.044). Multivariate analysis of the matched patients revealed that the TNM stage and preoperative CRP level were independent prognostic factors for OS.

**Conclusions:**

A high preoperative CRP level (> 5.0 mg/mL) predicts worse survival prognosis in patients who have undergone curative resection for esophageal squamous cell cancer.

## Introduction

Esophageal cancer (EC) is a common cause of cancer-related deaths worldwide [1], and almost 50% of patients with EC live in China, where the most common histological type of this cancer is esophageal squamous cell carcinoma (ESCC) [2]. Complete surgical resection is the best treatment for patients with EC [3]. EC is generally diagnosed in the later stage and progresses rapidly; therefore, the overall survival (OS) rate of EC remains poor [4–6]. Thus, it is necessary to identify the predictive factors of prognosis in EC patients. Several markers have been reported for predicting the prognosis of this disease [7–10]; however, none has been widely used in clinical practice.

It is being increasingly acknowledged that the intrinsic properties of tumor cells and the host inflammatory response determine the spread of tumors. C-reactive protein (CRP) is one of the acute-phase nonspecific proteins synthesized by the hepatocytes and regulated by interleukin-1, interleukin-6, and tumor necrosis factor (TNF) [11, 12]. Several reports have shown that a high preoperative serum CRP level is a disease-independent prognostic factor in a variety of tumors, such as those found in gastric cancer, lung cancer, renal cancer, and ovarian cancer [13–17]. Moreover, there are reports on a relationship between preoperative serum CRP levels and the prognosis of EC [18–22]. However, these studies were conducted on a small number of patients, and the imbalances between the groups in these retrospective analyzes may also have affected the results, resulting in indefinite conclusions. Owing to the inconsistent results, the role of serum CRP in EC remains controversial.

The present study aimed to test the relationship between preoperative serum CRP levels and EC-specific survival in patients who had undergone curative resection for esophageal squamous cell cancer. In order to arrive at a reliable conclusion, we ensured a good sample size (*n* = 961) in our study. The large sample size enabled us to perform propensity score analysis that allows matching of multiple factors to create similar groups for analysis. In this study, we performed a propensity-matched analysis of patients with high CRP levels and those with low CRP levels to identify the prognostic value of preoperative CRP levels.

## Materials and methods

### Patients

We conducted a large-scale retrospective study by searching the EC database at the Department of Thoracic Surgery, Sun Yat-sen University Cancer Center, Guangzhou, China. Clinical and pathological data were extracted from the medical records. Baseline data included information on age, sex, consumption of alcohol and smoking, and preoperative serum CRP levels. We also examined the surgical conditions and tumor biological features, including surgical incisions, intraoperative procedures, type of surgery, postoperative stage, tumor invasion depth, lymph node metastasis (N status), and tumor grade. The tumors were staged as per the American Joint Committee on Cancer (AJCC) Staging Manual (7th edition) [23]. For patients with EC, only tumor-lymph node-metastasis (TNM) stage I–III tumors were considered eligible for radical surgical resection and were included in this study. No patients had received neoadjuvant radio- or chemotherapy [24], while those who had received postoperative radio- and/or chemotherapy were included. Patients with inflammatory diseases that influenced the preoperative serum CRP levels, such as infections and collagen disease, and those with primary cancers in other organs were excluded.

We enrolled 961 patients with esophageal squamous cell carcinoma who had undergone curative esophagectomy between 2006 and 2012 at the Department of Thoracic Surgery, Sun Yat-sen University Cancer Center. Patients were followed up until death or until study completion. Only patients who survived for at least 60 days postoperatively were enrolled. The median follow-up duration was 24 months, and the maximum follow-up duration was 67 months. Routine preoperative laboratory examinations were performed, including immunoturbidimetry, to determine the serum CRP levels. All the patients were terminally diagnosed at the pathological department at Sun Yat-sen University Cancer Center. The tumors were pathologically staged as per the AJCC Staging Manual (7th edition). However, because the data were collected over several years, some patients were staged according to the 6th edition before the 7th edition was published in 2009. We recorded the exact locations, invasion depths, positive lymph nodes, metastases, and tumor grades in detail after the surgery. These data were used to compute the location as well as the T, N, and M stages of the tumor and subsequently stage the tumor according to the 7th edition.

The institutional review board of the Sun Yat-sen University Cancer Center approved the present study, and the study was approved by the ethics committee of Sun Yat-sen University Cancer Center (Ethical approval number: YB2016–070).

### Analysis method

A preoperative CRP level of > 5.0 mg/mL was considered high and was established as the cutoff (5.0 mg/mL) level using the professional X-tile program (version 3.6.1, copyright Yale University 2003–05). Differences between the two groups of patients were compared using the chi-square test. Three- and 5-year survival rates were calculated using life table analysis. We compared the survival rates of patients with high and low preoperative CRP levels using Kaplan–Meier analysis and generated survival curves. The log-rank test was used to test the significance of the differences between the high- and low-CRP level groups. Multivariate survival analysis was performed to derive a final model of the variables exhibiting independent significant relationships, including all the covariates that were significant in the univariate analysis, with survival rate. All the analyzes were performed using SPSS (version 22.0, IBM SPSS, Inc.). A two-sided *p* value of < 0.05 was considered statistically significant.

Propensity score matching (PSM) was performed to reduce the bias due to age, TNM stage, and tumor grade. One-to-one matching without replacement was performed using a caliper-match algorithm, with the caliper width set to 0.05 times the standard deviation of the logit of the propensity score. The quality of matching was assessed by comparing the standardized differences between the treatment groups, with a threshold of 0.1 indicating good balance between the groups. This procedure was performed using STATA 12.0 (version 12.0, Stata Corp, College Station).

## Results

Total 961 patients (749 men and 212 women; mean age, 58 years; range, 28–88 years) were enrolled. The preoperative serum CRP levels were > 5.0 mg/mL (mean, 18.92 mg/mL; median, 9.13 mg/mL; standard deviation, 31.25 mg/mL; range, 4.50–290.81 mg/mL) in 250 patients and ≤ 5 mg/mL (mean, 1.54 mg/mL; median, 1.25 mg/mL; standard deviation, 1.09 mg/mL; range, 0.06–4.48 mg/mL) in 711 patients.

**Fig. 1:**
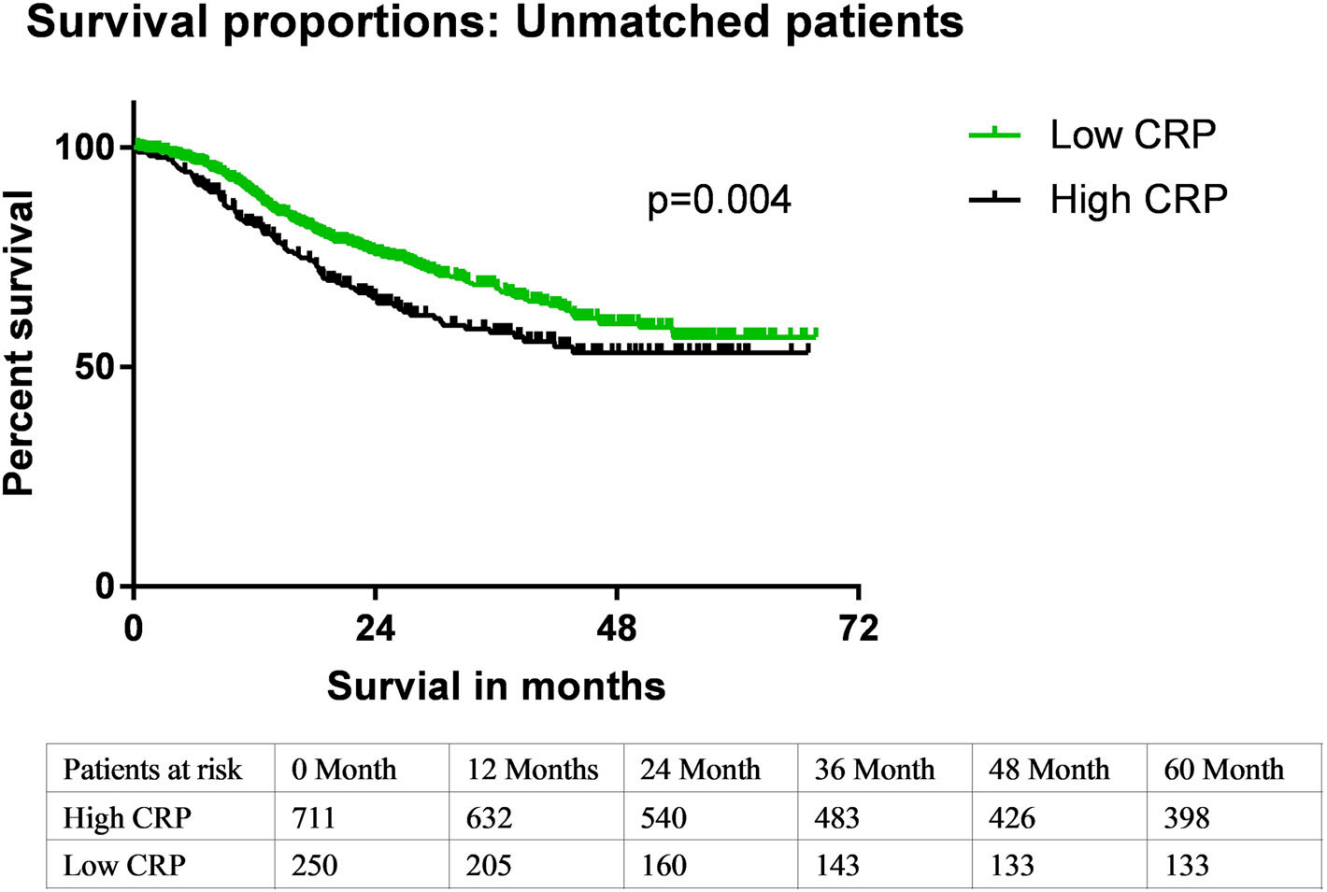
Kaplan-Meier–adjusted survival in postoperative esophageal squamous cell carcinoma patients. Overall survival of matched groups is shown

The clinicopathological characteristics of the patients are shown in Table 1. Before matching, there were significant differences in the distribution of age and the pathological T stage between patients with high preoperative CRP levels (> 5.0 mg/mL) and those with low preoperative CRP levels (≤ 5.0 mg/mL). Three- and 5-year survival rates were 68 and 56%, respectively, in patients with low preoperative CRP levels and 57 and 53%, respectively, in those with high preoperative CRP levels. After PSM, 176 patients with a low CRP level and 176 patients with a high CRP level were enrolled. There were no significant differences in the distribution of the clinicopathological characteristics between patients with high preoperative CRP levels (> 5.0 mg/mL) and those with low preoperative CRP levels (≤ 5.0 mg/mL). In the matched cohort, the differences in survival between the low- and high-CRP group remained significant (*p* = 0.044, Fig. 1); the 3-year and 5-year survival rates were 56 and 50%, respectively, in patients with high preoperative CRP levels (> 5.0 mg/mL) and 68 and 56%, respectively, in those with low preoperative CRP levels (≤ 5.0 mg/mL).

**Table 1:** Clinicopathological features of the unmatched and propensity scored-matched patients

	Unmatched			Matched		
	Low CRP(*n* = 711)	High CRP(*n* = 250)	*P*-value	Low CRP(*n* = 176)	High CRP(n = 176)	*P*-value
Gender						
Male	546 (76.7%)	203 (81.2%)	0.137	136 (77.3%)	141 (80.1%)	0.517
Female	165 (23.3%)	47 (18.8%)		40 (22.7%)	35 (19.9%)	
Age, y	58 (49,65)	60 (50,70)	0.001	60 (57–63)	60 (57–63)	0.735
Smoking						
Yes	455 (64%)	173 (69.2%)	0.137	112 (63.6%)	121 (68.7%)	0.312
No	256 (36%)	77 (30.8%)		64 (36.4%)	55 (31.3%)	
Drinking						
Yes	238 (33.5%)	95 (38.2%)	0.182	62 (35.2%)	70 (39.4%)	0.417
No	473 (66.5%)	155 (61.8%)		114 (64.8%)	106 (60.6%)	
Tumor site						
Cervix	17 (2.4%)	1 (0.4%)		6 (3.4%)	1 (0.6%)	0.777
Up	104 (14,6%)	40 (16.0%)	0.635	22 (12.5%)	31 (17.7%)	
Middle	350 (49.3%)	123 (49.2%)		94 (53.4%)	83 (47.4%)	
Low	240 (33.7%)	86 (34.4%)		54 (30.7%)	60 (34.2%)	
TNM stage						
IA	6 (0.8%)	3 (1.2%)		3 (1.7%)	0	0.970
IB	94 (13.2%)	17 (6.8%)		13 (7.4%)	18 (10.2%)	
IIA	117 (16.5%)	44 (17.6%)	0.254	30 (17.0%)	32 (18.2%)	
IIB	194 (27.3%)	69 (27.6%)		44 (25.0)	42 (23.9%)	
IIIA	158 (22.2%)	68 (27.2%)		47 (26.7%)	44 (25.0%)	
IIIB	83 (11.7%)	35 (14%)		26 (14.8%)	23 (13.1%)	
IIIC	59 (8.3%)	14 (5.6%)		13 (7.4%)	17 (9.7%)	
Differentiation						
Well	192 (27%)	86 (34.4%)		57 (32.4%)	63 (35.3%)	0.823
Moderate	404 (56.8%)	120 (48.0%)	0.086	85 (48.3%)	78 (44.3%)	
Poor	115 (16.2%)	44 (17.6%)		34 (19.3%)	36 (20.5%)	
Surgical incision						
Left	434 (61.0%)	165 (66.0%)	0.176	105 (59.7%)	117 (66.5%)	0.199
Right	277 (39.0%)	85 (34.0%)		71 (40.3%)	59 (33.5%)	

Abbreviations: *CRP* C-reactive protein

**Fig. 2:**
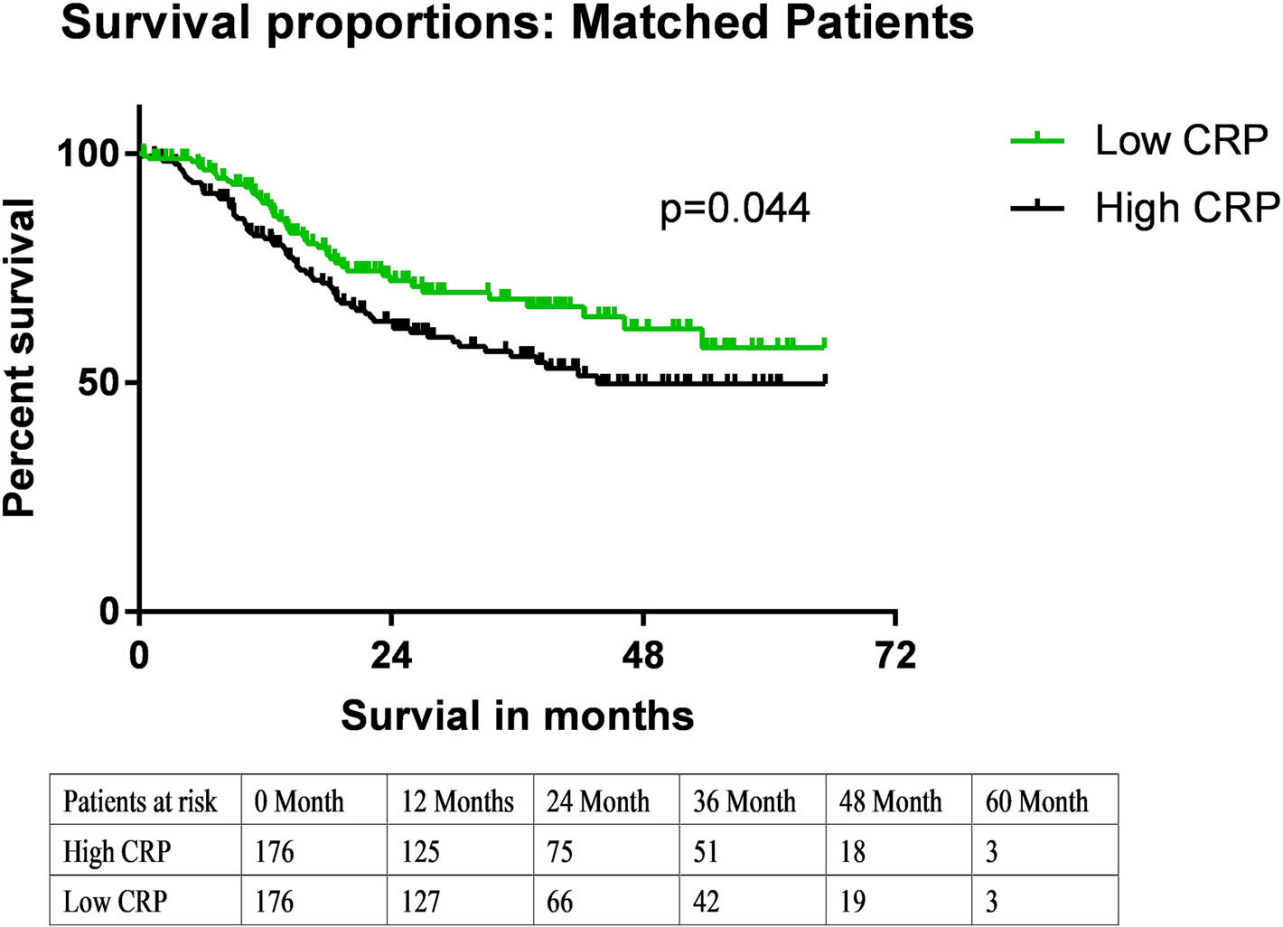
Kaplan-Meier–adjusted survival in postoperative esophageal squamous cell carcinoma patients. Overall survival of unmatched groups is shown

Before matching, univariate analysis showed that age (*p* < 0.001), TNM stage (*p* = 0.001), history of alcohol consumption (*p* = 0.024), tumor grade (*p* = 0.039), and preoperative serum CRP levels (*p* = 0.008) were significant risk factors for postoperative survival. Survival curves were used to illustrate the differences in the OS duration between patients with high and low preoperative CRP levels (Fig. 2). After matching, the univariate analysis showed that age (*p* < 0.001), TNM stage (*p* < 0.001), tumor site (*p* < 0.001), and preoperative serum CRP levels (*p* = 0.044) were significant risk factors for postoperative survival. Moreover, surgical incision was not a significant risk factor (*p* = 0.140). The factors mentioned above were included in the multivariate Cox regression analysis. The result showed that the TNM stage and the preoperative CRP level were independent prognostic factors for OS (Table 2).

**Table 2:** Univariable and Multivariable Cox Regression for overall survival After Propensity Score Matching

Variables	Univariable Analysis	Multivariable Model			
	*p*-value	*p*-value	Exp(B)	95% CI for Exp(B)	
Age	< 0.001	0.084			
Smoking history	0.717				
Drinking history	0.395				
CRP level	0.044	0.022	0.646	0.443	0.942
Tumor site	< 0.001	0.087			
Surgical incision	0.140	0.295			
Tumor grade	0.734				
TNM stage	< 0.001	< 0.001	1.485	1.299	1.699

## Discussion

In China, the most common type of EC is squamous cell carcinoma. Therefore, our study focused on the prognosis of patients with this type of tumor. It has been reported that some factors may affect the prognosis of patients with EC, such as patient status; tumor biological behavior, including the number of cancer-positive lymph nodes; histopathological cell type; histological grade; cancer location, including the esophagogastric junction; genetic mutations; and surgery type. However, no preoperative markers that can predict the prognosis of esophageal squamous cell carcinoma are currently widely used in clinical practice.

CRP is a representative reactant of acute and chronic phase inflammation. A link between inflammatory reactions and cancer has been reported by Virchow in 1863 that identified leukocyte infiltration in neoplastic tissues and suggested that these sites of chronic inflammation were the origin of the cancer [25]. There have also been reports of a relationship between inflammation and cancer progression [26]. It has now been widely accepted that the elevation in the preoperative serum CRP levels is a reliable indicator of poor postoperative prognosis in patients with certain malignancies, including those of the lung, kidney, ovary, and gastrointestinal tract. Recently, Ibuki et al. showed an association of postoperative CRP levels with poorer OS and recurrence-free survival [27, 28].

Some studies have reported a relationship between the prognosis of EC and preoperative CRP levels. However, these studies did not demonstrate a convincing relationship between these two parameters.

The 2001 study by Nozoe et al. included 262 EC patients and showed that CRP can be an independent biomarker in patients with EC [22]. Thereafter, in 2003, Ikeda et al. gave the same conclusion based on their retrospective analyzes of the clinicopathological factors in 356 patients wherein they aimed to identify factors related to prognosis [29]. Gockel et al. investigated 291 EC patients who underwent curative resection and concluded that a high level of CRP (≥ 5 mg/dL) is associated with tumor progression and poor OS [21]. The study by Wang et al. that included 123 patients showed that the pretreatment serum levels of CRP and albumin can be used to predict survival in EC patients treated with radiotherapy [30]. Furthermore, the preoperative CRP level can also play the same role in patients with small carcinoma of the esophagus or esophageal adenocarcinoma and in patients with early stage esophageal squamous cell carcinoma [19, 31, 32]. The report from Shimada et al. included patients with metastatic disease at the time of surgery and showed that the preoperative serum CRP levels could be a prognostic indicator in patients with esophageal squamous cell carcinoma [18]. A recent study from Otowa et al. showed that preoperative CRP level is a prognostic factor for cStage III EC [33]. All these studies showed that preoperative CRP level can be a significant prognostic indicator in EC patients.

However, some contradictory reports also exist. The report by Zingg et al. showed significantly better survival in patients with normal CRP compared to that in patients with raised CRP levels among those who received neoadjuvant therapy that comprised 2 cycles of 5-fluorouracil (5-FU) and cisplatin in combination with 45–54 Gy of radiotherapy [34]. Owing to the inconsistent results, the role of serum CRP in EC remains controversial.

In addition, the sample size in previous studies was generally low. For example, Shimada et al. included 150 patients in the study, and only 35 patients were grouped into the high-CRP group. Similarly, Crumley et al. analyzed 120 patients; of these, only 15 exhibited high preoperative serum CRP levels. Therefore, the statistical power of these studies was weak because of the small number of patients. Owing to the limitation presented by the relatively smaller sample size, no study has performed PSM analysis to study the prognostic effect of CRP level. In our study, we included more patients (961) than those in previous studies. Moreover, PSM was performed to reduce the potential confounders. Before matching, the patients who exhibited high preoperative serum CRP levels had worse OS than those with low preoperative serum CRP levels (*p* = 0.004). In the matched cohort, the survival in the low-CRP group was better than that in the high-CRP group (*p* = 0.044). Additionally, multivariate Cox regression analysis of the matched patients showed that the CRP level was an independent prognostic factor for OS. As per the result, we concluded that the preoperative serum CRP level was an independent prognostic factor for patients with esophageal squamous cell carcinoma, a finding that is consistent with some previous reports.

Many factors, such as age and nutrition status, can affect the preoperative serum CRP levels [35]. By using PSM, the factors that affect CRP level can be balanced between the groups to some extent. In our study, the patients were well matched for age, tumor grade, and TNM stage. Univariate analysis showed that these three cofactors were significant risk factors for postoperative survival. After PSM, the difference between the two groups disappeared. Balanced grouping confirmed the conclusion. Another advantage of PSM is that after one-to-one matching, the high- and low-CRP groups had the same sample size.

Recently, some studies focus on the prognostic value of postoperative CRP level, and got some interesting results. In 2017, Ibuki et al. draw conclusion that the high postoperative CRP level can predict bad survival of patients who received esophagectomy [27]. Furthermore, this indicator can also predict the survival of patients with other carcinomas. In patients received non-small cell lung cancer resection, the research proposed by Shinohara showed that elevated 6-week postoperative C-reactive protein were related to worse OS [36]. The study by Pastorino et al. got a conclusion that the immediately elevated CRP level (postoperative day 3, 5, and 7) were related to worse outcome after surgery [37]. Furthermore, these studies indicate the predicative potential of postoperative serum CRP level, and the combination of preoperative and postoperative CRP may improve OS prediction in esophageal cancer patients. But in our study, we don’t collect the data of the postoperative CRP level. Maybe we can pay more attention to this aspect in further research.

The present study has certain limitations. Although we attempted to offset the potential bias between the low- and high-CRP groups with PSM, the remaining bias may have affected the results.

## Conclusion

In conclusion, after a careful comparison and a propensity score matching analysis, we found that the A high preoperative CRP level (> 5.0 mg/mL) predicts worse survival prognosis in patients who have undergone curative resection for ESCC.

## Data Availability

Please contact author for data requests.

## Abbreviations

AJCC: American Joint Committee on Cancer
CRP: C-reactive protein
EC: Esophageal carcinoma
ESCC: Esophageal squamous cell carcinoma
OS: Overall survival, Lymph node status and metastasis
PSM: Propensity score matching
TNM: Tumor invasion depth,
